# Gas-Phase Fragmentation of Oligoproline Peptide Ions Lacking Easily Mobilizable Protons

**DOI:** 10.1007/s13361-013-0585-1

**Published:** 2013-04-23

**Authors:** Magdalena Rudowska, Robert Wieczorek, Alicja Kluczyk, Piotr Stefanowicz, Zbigniew Szewczuk

**Affiliations:** Faculty of Chemistry, University of Wrocław, Wrocław, Poland

**Keywords:** Quaternary ammonium salts, Charge-tagged peptides, Proline fragmentation, Charge-remote fragmentation, Mobile protons, HDX

## Abstract

The fragmentation of peptides containing quaternary ammonium group, but lacking easily mobilizable protons, was examined with the aid of deuterium-labeled analogs and quantum-chemical modeling. The fragmentation of oligoproline containing quaternary ammonium group involves the mobilization of hydrogens localized at α- and γ- or δ-carbon atoms in the pyrrolidine ring of proline. The study of the dissociation pattern highlights the unusual proline residue behavior during MS/MS experiments of peptides.

**Electronic supplementary material:**

The online version of this article (doi:10.1007/s13361-013-0585-1) contains supplementary material, which is available to authorized users.

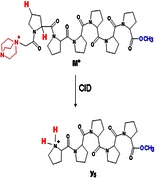

## Introduction

Mass spectrometric characterization of the compounds relies on the analysis of structurally significant fragment ions, formed during fragmentation of precursor ions [1]. For protonated peptides, the main fragmentation mechanism, called charge directed, is based on the presence of mobile proton, which can migrate along the peptide backbone [2–4]. The protonation of amide nitrogen weakens the peptide bond, resulting in peptide fragmentation after gas-phase collisional activation [5]. However, in the presence of basic amino acid residue such as arginine, lysine, or histidine, the mobile proton could be sequestered, preventing charge directed fragmentation.

The alternative mechanism, called charge remote, was first observed during the study of fatty acids [6] and is caused by the intramolecular hydrogen shifting within the precursor ion [7–10]. In peptides lacking easily mobilizable protons, there are three possible mobilization pathways involving protonated guanidine group of arginine, C-terminal carboxyl, and peptide backbone amidic protons transfer [11, 12].

The effect of the arginine residue can be mimicked by peptide derivatization with a fixed positive charge-carrying molecule [13, 14], including quaternary ammonium salts (QAS) [15–18], which enables MS analysis without proton transfer from solvent. In this case, there are three types of hydrogens (α, β and amide) that could undergo intramolecular migration to yield fragment ions. In a previous study, it was proposed that the charge remote fragmentation involves the amide hydrogen shifting [7], which was then confirmed by the analysis of selective deuterium labeled analogs [8]. In peptides containing proline, a residue without an amide hydrogen, β-hydrogen shifting was also observed [7].

The aim of our work was to investigate fragmentation pathways of peptides lacking easily mobilizable protons. For this purpose, we designed and synthesized a series of oligoproline peptides derivatized with QAS. We performed ESI-MS/MS experiments on M^+^ ions of all the synthesized QAS-peptide derivatives and, with the aid of base-catalyzed hydrogen/deuterium exchange, described by us previously [19], we examined the charge remote fragmentation mechanism, using collision-induced dissociation (CID).

## Experimental

### Materials

All solvents and reagents were used as supplied. Fmoc-Pro-OH was purchased from Novabiochem (Darmstadt, Germany). Boc-Pro-OH and Boc-Pro-Merrifield were synthesized in-house. Fmoc-Sar-OH was obtained according to the procedure described by Paquet [20]. The Merrifield support (2 % cross-linked chloromethylated copolymer of styrene-divinyl benzene) was purchased from Reanal (Budapest, Hungary), and esterificated with Boc-Pro-OH in the presence of cesium carbonate [21]; the loading capacity was calculated according to the mass difference and was in the range of 0.6–0.7 mmol × g^–1^. The MBHA-Rink amide resin (0.69 mmol/g), 2-(1*H*-benzotriazole-1-yl)-1,1,3,3-tetramethylaminium tetrafluoroborate *N*-oxide and trifluoroacetic acid (TFA) were purchased from Iris Biotech (Marktredwitz, Germany). Triethylamine (Et_3_N) and 1,4-diazabicyclo[2.2.2]octane (DABCO), used for QAS formation, and solvents for peptide synthesis (*N,N*-dimethylformamide, dichloromethane, tetrahydrofuran (THF), and *N*-ethyldiisopropylamine) were obtained from Sigma Aldrich (St. Louis, MO, USA); iodoacetic acid from Merck; diisopropylcarbodiimide and triisopropylsilane from Fluka (Buchs, Switzerland). Deuterium oxide (D_2_O, 99.9 % purity) was obtained from Cambridge Isotope Laboratories (Andover, MA, USA), Inc.

### Peptide Synthesis

Solid phase peptide synthesis was performed manually in polypropylene syringe reactors (Intavis AG, Koeln, Germany) equipped with polyethylene filters, according to a standard Boc or Fmoc procedure, respectively.

### QAS Formation

Peptides containing 2-(triethylammonium)acetyl and 2-(4-aza-1-azoniabicyclo[2.2.2]octylammonium)acetyl were synthesized according to the procedure described by us previously [14]. The N-terminal amino group of peptides attached to the resin was iodoacetylated for 3 h in the presence of *N,N′*-diisopropylcarbodiimide followed by treatment with a 5-fold excess of tertiary amine (Et_3_N and DABCO, respectively) for 24 h at room temperature (Supplementary Data, Figure 1S, procedure A).

#### C-Terminal Amide Formation

Cleavage of the derivatized peptides from the MBHA-Rink amide resin was accomplished using a solution of TFA/H_2_O/triisopropylsilane (95/2.5/2.5, vol/vol/vol) at room temperature for 2 h. Peptides were precipitated from the cleavage mixture with ice-cold diethyl ether (Et_2_O) (Supplementary Data, Figure 1S, procedure B).

#### C-Terminal Methyl Ester Formation

Cleavage of the derivatized peptides from the Merrifield resin was accomplished using a solution of Et_3_N/THF/MeOH (1/2/2, vol/vol/vol) according to the modified procedure described by Dondas et al. [22]. A sample of the peptidyl resin (5 mg) was placed in a standard CEM glass microwave vial and swelled in THF (0.5 mL) for 1 h. Then the solution of the Et_3_N/THF/MeOH (1 mL) was added. The vial was transferred into the microwave synthesizer cavity and subjected to microwave irradiation with a gas cooling (pressure of 20 psi was maintained during irradiation) for a 15 min at 70 °C with magnetic stirring. The resin was removed by filtration and rinsed with THF. The combined filtrates were evaporated in a stream of nitrogen. Crude compounds were dissolved in water, lyophilized, and purified by reversed phase HPLC (Supplementary Data, Figure 1S, procedure C).

All the microwave-assisted reactions were carried out in a monomode microwave Discover CEM apparatus (CEM Corporation, Matthews, NC, USA), equipped with IR temperature sensor and gas cooling system. The reactor was used in “standard” mode (a set temperature value was applied for a specific time).

### Purification

Because of the scale of synthesis, all QAS-peptide derivatives were purified by the analytical HPLC using a Thermo Separation HPLC system with a UV detection (210 nm) on a Vydac Protein RP C18 column (4.6 × 250 mm, 5 μm), with a gradient elution of 0–40 % B in A (A = 0.1 % TFA in water; B = 0.1 % TFA in acetonitrile/H_2_O, 4:1) for 30 min (flow rate 1 mL/min). The main peak, corresponding to the QAS-peptide derivative, was collected and the fraction was lyophilized.

### Isotopic Exchange

H/D exchange was initiated by dissolving 0.1 mg of QAS-peptide derivative in 200 μL of D_2_O with the addition of 2 μl of Et_3_N. After 2 min, two hydrogens at α-carbon atom bound to QAS were exchanged by deuteriums, as judged from the ESI-MS/MS analysis. For QAS-peptides containing C-terminal amide group two additional hydrogens were exchange by deuteriums.

### Mass Spectrometry

Mass spectrometric experiments were performed on a Bruker micrOTOF-Q mass spectrometer (Bruker Daltonics, Bremen, Germany) and on an FT-ICR (Fourier transform ion cyclotron resonance) Apex-Qe Ultra 7 T instrument (Bruker Daltonics), equipped with an ESI source. Spectra were recorded using three different solvent systems: (1) 50:50 acetonitrile–water mixture containing 0.1 % HCOOH, (2) H_2_O containing 1 % Et_3_N, D_2_O, and (3) D_2_O containing 1 % Et_3_N. Analyte solutions were infused at a rate of 3 μL/min. The instruments were operated in the positive ion mode and calibrated before each analysis with the Tunemix mixture (Bruker Daltonics) in quadratic method. The instrument parameters were as follows: scan range: 100–1000 *m/z* and 200–1600 *m/z* (TOF and FT-ICR instruments, respectively); drying gas: nitrogen, flow: 4.0 L/min; nebulizer gas: nitrogen, flow rate: 1.5 L/min; temperature: 200 °C; potential between the spray needle and the orifice: 4.2 kV. In the MS/MS experiments, the singly charged M^+^ precursor ions were selected on the quadrupole and subsequently fragmented in the collision cell. Argon was used as a collision gas. The obtained fragments were directed to the mass analyzer and registered as an MS/MS spectrum. The collision energy was optimized for the best fragmentation pattern at range 10–40 eV. For deuterated compounds, a narrow isolation window (± 0.1 Da) was used to select monoisotopomeric peaks only. For MS spectra analysis, a Bruker Compass DataAnalysis 4.0 software was used. A Bruker sophisticated numerical annotation procedure (SNAP) algorithm was used for finding peaks. The SNAP algorithm calculates the isotopic distribution of a given mass, charge, and mean molecular constitution, and uses this isotopic pattern for a nonlinear fit. This fit delivers the monoisotopic mass and the line shape parameters of the pattern [23]. All obtained signals had a mass accuracy error in the range of 2 ppm.

To establish fragmentation patterns of primary fragments, a series of MS^3^ experiments were performed. The in-source CID (ISCID) energy was set at 150 eV to induce in-source fragmentation of the compounds prior to entering the quadrupole. The quadrupole collision energy was set at 25 eV in order to generate product ions while ensuring that the precursor ion remained abundant.

### Calculation Details

Molecular orbital studies on structure and stability of **(d**_**4**_ **-NH,C** ^**α**^ **H)-4b**, [*b*_*2*_ – DABCO] and [*a*_*3*_ – DABCO] have been done on DFT level of theory. All calculations were performed with the Gaussian 09 [24] suite of programs using the hybrid functional B3LYP and d95(d,p) basis set. Presented structures show fully DFT optimized structures of investigated compounds.

Quantum chemical calculations were performed for two pathways of **4b** precursor fragmentation that can lead to [*b*_*2*_ – DABCO] and [*a*_*3*_ – DABCO] ions depending on presence of mobile proton. For both reactions DFT predicts presence of stable products: [*b*_*2*_ – DABCO] and [*a*_*3*_ – DABCO] ions.

## Results and Discussion

We investigated charge remote fragmentation pathways of QAS derivatized peptides, lacking easily mobilizable protons. To eliminate amide hydrogens, a series of model peptides containing three to six proline residues was synthesized. The N-terminal amino groups of peptides were derivatized using either triethyl- or 4-aza-1-azoniabicyclo [2.2.2]octylammonium acetyl moiety, which introduced a stable positive charge to the peptides and enabled MS analysis without proton transfer from a solvent. To prevent mobilization of a proton from the carboxyl group the C-termini of peptides were modified by amide or methyl ester group formation. The sequences and molecular masses of all the synthesized QAS-peptides are presented in Table 1S.

### ESI-CID-MS/MS Analysis of Proline QAS-Peptide Derivatives

Tandem mass spectra of protonated polyproline or proline-rich peptides have been studied extensively because of the known influence of proline residue on the dissociation pattern [25–27]. The formation of *y*-type ions from backbone cleavage at the N-terminal side of proline is often the most dominant fragmentation pathway, which was explained by high proton affinity of the proline residue and unfavorable structure of *b*-type ions [28–30]. Of the amino acids with non-basic side chains, proline has the highest gas-phase proton affinity [31]. When involved in a peptide bond, the amide nitrogen of proline residue is tertiary and, thus, more basic than the corresponding secondary nitrogen atoms of the other common amino acid residues [32]. This, therefore, encourages protonation on the nitrogen of proline residue and will lead to a high yield of *y*-type fragments from backbone cleavage at the N-terminal side of proline [24]. However, a recent computational study showed that unlike proposed earlier in the literature, there is only a small destabilization attributable to strain in the bicyclic *b*-type ion, and these types of fragments are formed in fragmentation of protonated peptides [33].

**Figure 1:**
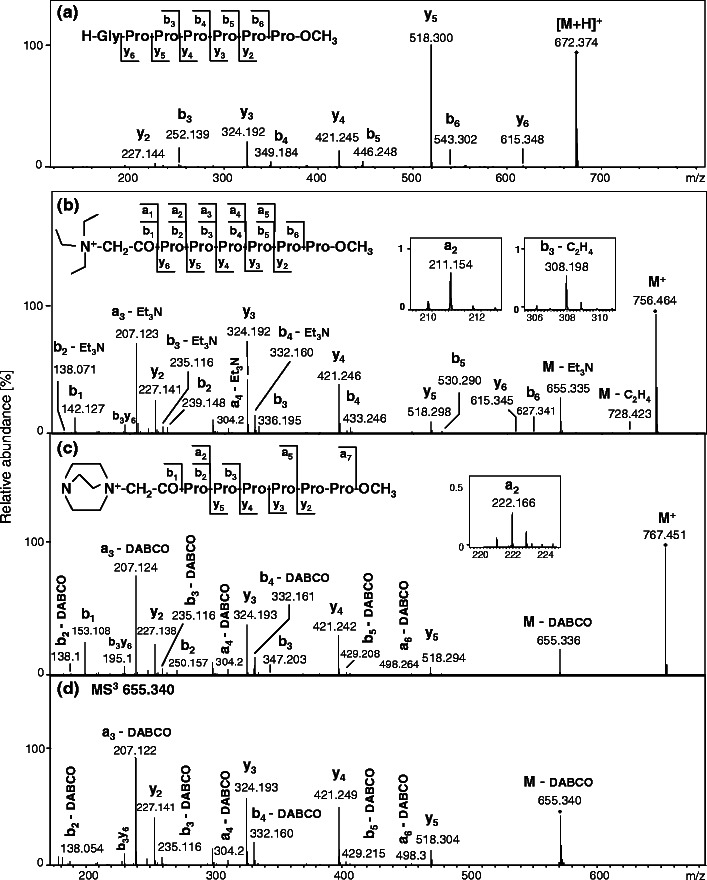
ESI-MS/MS spectra of the [M + H]^+^ ion of peptide H-Gly-(Pro)_6_-OCH_3_ (**a**), M^+^ molecular ions of peptides: **8a** (**b**) and **8b** (**c**), and MS^3^ spectrum of [M – DABCO] fragment ion from the spectrum C (**d**). For peptides **8a** and **8b**, the peaks of representative fragments *a* and [*b* – C_2_H_4_], respectively, are shown in insets. The collision energy was set at 13 eV (**a**), 35 eV (**b**), 37 eV (**c**), and 25 eV (**d**)

To verify the influence of proline residue on the peptide fragmentation pathway, we synthesized a peptide H-Gly-(Pro)_6_-OCH_3_ and analyzed it by ESI-MS/MS. The fragmentation of the protonated peptide reveals a series of *y*-type ions, ranging from *y*_*2*_ to *y*_*6*_, and the accompanying *b*-type ions (Figure 1a), which is in agreement with the results obtained previously and suggests that the bicyclic *b*-type ions may be formed.

Fragmentation of derivatized peptides with fixed charge on their N-termini leads usually to *a*- and *b*-type fragment formation [14]. Surprisingly, the analysis of the MS/MS spectra of peptides **8a** and **8b** (Figure 1b, c, respectively) reveal the most abundant series of *y*-type ions, ranging from *y*_*1*_ to *y*_*5*_, and *y*_*6*_ for triethylammoniumacetyl peptide derivative. The corresponding *b*-type ions were also observed. The quaternary ammonium derivative of acetic acid residue (R_3_N^+^-CH_2_-CO-) was considered as the first amino acid in the peptide sequence. The fragmentation showed also a very low intensity *a*-type fragment ions (Figure 1b, c, insets) and a series of *a*- and *b*-type ions with neutral losses as the consequence of elimination of a tertiary amine (101.120 for triethylamine or 112.100 for DABCO). In the fragmentation spectra, the most abundant ions are [*a*_*3*_ – DABCO] and [*a*_*3*_ – Et_3_N], although *a*_*3*_ ions are rarely observed in CID of protonated peptides [34, 35]. The low intensity or absence of *a*_*3*_ fragments has been attributed in literature to the facile fragmentation to form *b*_*2*_ ion as well as elimination of ammonia molecule to form the [*a*_*3*_ – NH_3_] ion [28, 36]. In the case of oligoproline peptides lacking easily mobilizable hydrogens the mechanism of [*a*_*3*_ – DABCO] or [*a*_*3*_ – Et_3_N]^+^ ions formation is affected by the absence of mobile proton.

Theoretical research shows that the presence of mobile proton determines the product of fragmentation of **4b**. The mobile proton modifies the length of C–N + bond from 1.358 Å (without mobile proton) to 1.485 Å (with mobile proton). The position of the weakest bond determines the product of reaction presented on Scheme 4, [*b*_*2*_ – DABCO]. Upon decomposition in absence of mobile proton, a different product is suggested by quantum results. The analysis of C–C distance of backbone carbons related to bond strength shows that the rearrangement of electron density in absence of mobile proton reorganizes the bond strengths (the C–C bonds follow the pattern strong-weak-strong-strong [1.544 Å-1.555 Å-1.548 Å-1.543 Å]) and suggests [*a*_*3*_ – DABCO] cation as the preferred product of decomposition (Supplementary Data, Figure 13S).

Triethylammoniumacetyl peptide derivative produces also [*a* – C_2_H_4_] and [*b* – C_2_H_4_] fragment ions (Figure 1b, inset), due to a partial QAS group fragmentation by Hofmann elimination, which was described by us in detail previously [37]. Similar fragmentation pathways were observed for other analyzed QAS-peptide derivatives **1a**-**b**–**7a**-**b**, both for C-terminal amide and methyl ester modification (Supplementary Data, Figures 2S–5S).

The MS^3^ fragmentation of [M – DABCO] ion produced a series of *y*-, [*a* – DABCO], and [*b* – DABCO] type ions, analogous to that observed in the MS/MS spectrum of peptide **8b** (Figure 1d). The obtained results may suggest that the fragmentation of QAS-peptides lacking easily mobilizable protons is initiated by QAS group elimination. This suggestion was confirmed by the analysis of MS/MS spectra of peptides **8a** and **8b**, obtained at various fragmentation energies (Supplementary Data, Figures 6S, 7S). For the energy of 20 eV, only a peak corresponding to fragment [M – QAS] was observed. The other fragment ions were produced in higher collision energies.

**Figure 2:**
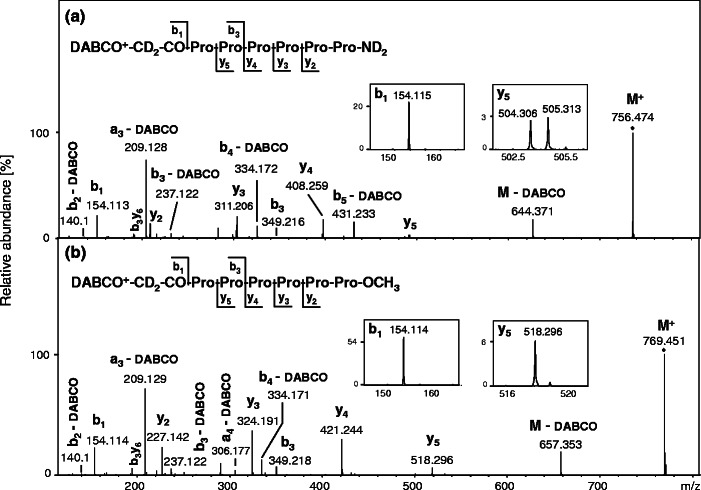
ESI-MS/MS spectra of the M^+^ molecular ions of deuterated peptides: **(d**_**4**_ **-NH,C** ^**α**^ **H)-4b** (**a**) and **(d**_**2**_ **-C** ^**α**^ **H)-8b** (**b**). The peaks of representative *b* and *y* fragments are shown in insets. The collision energy was set at 37 eV (**a**) and 38 eV (**b**)

To verify the mechanism of QAS-peptides fragmentation we performed the MS/MS experiments on a series of deuterated analogs of synthesized compounds with all exchangeable hydrogens replaced by deuteriums. The QAS-peptides **5a**-**b**–**8a**-**b** do not contain any mobile hydrogens (amide hydrogens, acidic side chains, free N- and C-terminal groups), which can be exchanged by deuteriums in D_2_O at neutral pH. However, we found previously that the hydrogens at α-carbon atom bound to QAS undergo H/D exchange in alkaline D_2_O solution via ylide formation [17]. This approach enabled the easy and fast introduction of two deuterium atoms into peptides **5a**-**b**–**8a**-**b**. For peptides **1a**-**b**–**4a**-**b**, four hydrogens were exchanged: two from α-carbon atom bound to QAS and two belonging to C-terminal amide group. The comparison of MS/MS spectra of peptides **4b** and **8b**, incubated in D_2_O with the addition of 1 % Et_3_N, is presented in Figure 2.

The fragmentation of M^+^ ion of QAS-peptides dissolved in alkaline D_2_O solution showed a series of N-terminal fragment ions with masses higher by 2 Da, compared with the corresponding fragments obtained in the mixture of H_2_O/MeCN/HCOOH, due to the exchange of relatively acidic hydrogens at α-carbon atom bound to QAS group into deuteriums [17]. For peptide **(d**_**2**_ **-C** ^**α**^ **H)-8b** the masses of C-terminal fragments remained unchanged, which indicates that introduced deuteriums are not mobile and do not shift during MS/MS experiments. For peptide **(d**_**4**_ **-NH,C** ^**α**^ **H)-4b** the exchange of two C-terminal amide hydrogens by deuteriums caused mass shifting of *y*-type ions by 2 Da. However, all of these ions (*y*_*2*_-*y*_*5*_) were represented by pairs of peaks, differing by one mass unit (Figure 2a, inset). These results suggest that during the fragmentation a mobile proton is generated, which can participate in a hydrogen scrambling with deuterons from C-terminal amide group. Similar effect was observed by us previously during fragmentation of deuterated triethylammoniumacetyl peptide derivatives [25]. In contrast, all N-terminal fragment ions of peptide **(d**_**4**_ **-NH,C** ^**α**^ **H)-4b** were represented by single peaks, which suggest that during this fragmentation the mobile proton is not generated and as a consequence, hydrogen scrambling does not occur. Analogous results were obtained for other tested deuterated QAS-peptide derivatives (Supplementary Data, Figures 8S–11S).

### Fragmentation Pathways of Oligoproline QAS-Peptides

The proposed mechanisms are presented for one representative peptide **(d**_**4**_ **-NH,C** ^**α**^ **H)-4b**.

#### y-Type Ion Formation

The MS/MS analysis of all synthesized QAS-peptide derivatives indicates that *y*-type ion formation occurs according to a mechanism similar to b_n_-y_m_ fragmentation pathway [3]. It is initiated by QAS group elimination, since in the MS/MS spectra recorded at low collision energies only a signal corresponding to the fragment [M – QAS] was observed (Supplementary Data, Figure 7S). In addition, MS^3^ of a fragment [M – QAS] generated a series of *y*-type ions, as well as *a*- and *b*-type fragment ions with the loss of QAS group (Figure 1d). Owing to the elimination of quaternary ammonium salt, a mobile proton is probably formed and participates in intramolecular exchange with deuterons located in the C-terminal amide group. This would confirm an earlier observation of Dong at al. [38] that the cleavage at N-terminal side of Pro is unfavorable when protons are non-mobile.

**Scheme 1:**
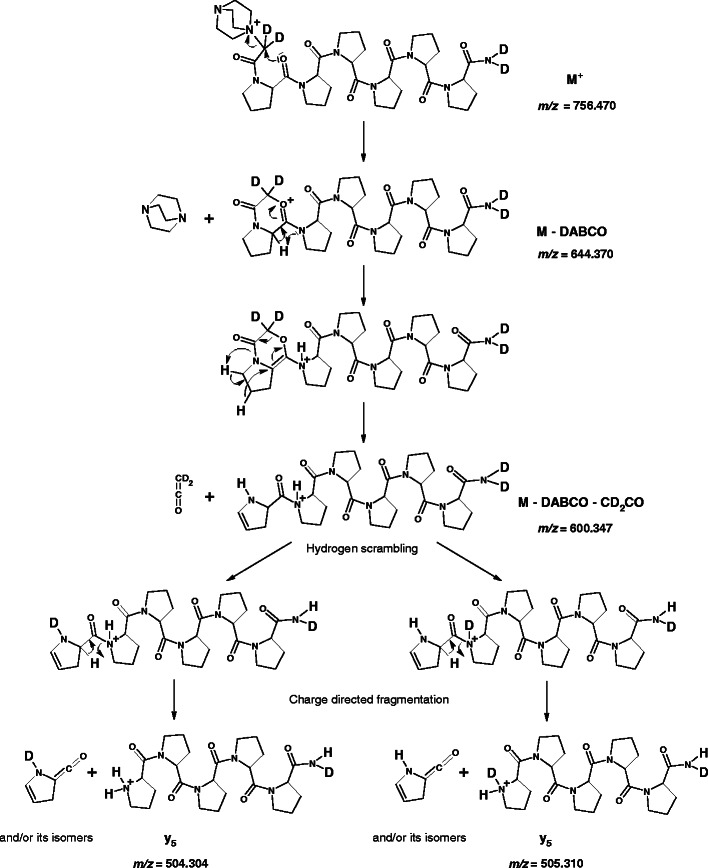
Possible pathways of *y*-type ions formation from **(d**_**4**_ **-NH,C** ^**α**^ **H)-4b** precursor ion, proposed on the basis of ESI-MS/MS spectrum presented in the Figure 2a. Theoretical *m/z* values are given

The QAS group elimination may be initiated by a nucleophilic attack of carbonyl oxygen atom at α-carbon atom bound to QAS group, which results in a neutral loss of DABCO molecule and a fragment [M – DABCO] formation (Scheme 1). A related fragmentation pathway was proposed previously for the fixed-charge sulfonium [39] and ammonium [40, 41] ion derivatives. The ensuing rearrangement and a shift of a proline residue α-hydrogen leads to a mobile proton generation. However, the *y*-type ions formation requires mobilization of one more proton, which can be one of the hydrogens located at δ-carbon atom from the cyclic side chain of proline residue. This mobile proton generation may occur as a consequence of ethenone elimination. The mobilization of δ hydrogen is accompanied by a shifting of the hydrogen located at γ-carbon atom. In the MS/MS spectrum of QAS-peptide **(d**_**4**_ **-NH,C** ^**α**^ **H)-4b**, recorded at low collision energy (25 eV), a very low intensity peak at *m/z* = 600.3 corresponding to a fragment [M – DABCO – CD_2_CO] could be observed (Supplementary Data, Figure 12S). Then a hydrogen scrambling may occur, since there are now two new mobilizable hydrogens in the peptide ion. The subsequent shift of the α-hydrogen from proline residue leads to two *y*_*5*_ ions that differ from each other by 1 Da, which results from the contribution of hydrogens and deuteriums. The proposed mechanism may explain the double peaks of *y*-type ions observed during **(d**_**4**_ **-NH,C** ^**α**^ **H)-4b** peptide fragmentation (Figure 2a, inset). For QAS-peptides with C-terminal methyl esters all *y*-type ions were represented by single peaks, since these peptides do not contain any deuterons, which may scramble with generated mobile protons.

#### a- and b-Type Ion Formation

**Scheme 2:**
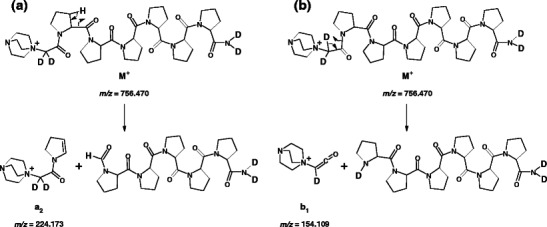
Possible pathways of a_2_ (**a**) and b_2_ (**b**) ions formation from **(d**_**4**_ **-NH,C** ^**α**^ **H)-4b** precursor ion, proposed on the basis of ESI-MS/MS spectrum presented in the Figure 2a. Theoretical *m/z* values are given

The major fragmentation pathway in the formation of *a*- and *b*-type ions involves a shift of the amide hydrogen, of the residue at which the cleavage occurs, to the neutral fragment of the peptide [16]. In the case of proline residue the most likely way to obtain *a*-type ions involves transfer of β-hydrogen from the proline cyclic side chain (Scheme 2a), whereas the formation of *b*-type ions is a result of a neighboring amide hydrogen shifting. Since oligoproline peptides do not contain amide hydrogens, it may be assumed that *b*-type ion are formed due to α-hydrogen shifting (Scheme 2b). The *m/z* value of b_1_ fragment (154.11) indicates accurately that one of the deuterons located at α-carbon atom of N-terminal DABCO-acetyl residue was transferred to the neutral C-terminal fragment.

**Scheme 3:**
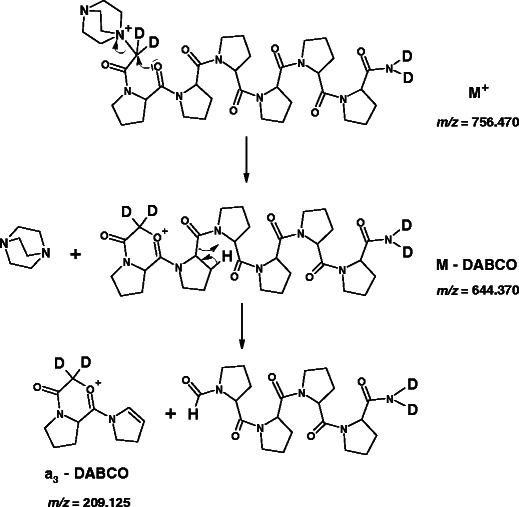
Possible pathway of [a_3_ – DABCO] ion formation from **(d**_**4**_ **-NH,C** ^**α**^ **H)-4b** precursor ion, proposed on the basis of ESI-MS/MS spectrum presented in the Figure 2a. Theoretical *m/z* values are given

A series of *a*- and *b*-type ions was accompanied by corresponding ions, where QAS group was eliminated as a tertiary amine. In both fragmentation pathways in the first step QAS group is eliminated, due to the nucleophilic attack of carbonyl oxygen at α-carbon atom bound to QAS group. The next step in [*a* – DABCO] ion formation involves a transfer of the proline β-hydrogen, according to the mechanism for *a*-type ion generation (Scheme 3). In the MS/MS spectra of deuterated QAS peptide derivatives these types of ions were represented by single peaks, which may suggest that the peptide bond dissociation occurs before the mobile proton formation.

**Scheme 4:**
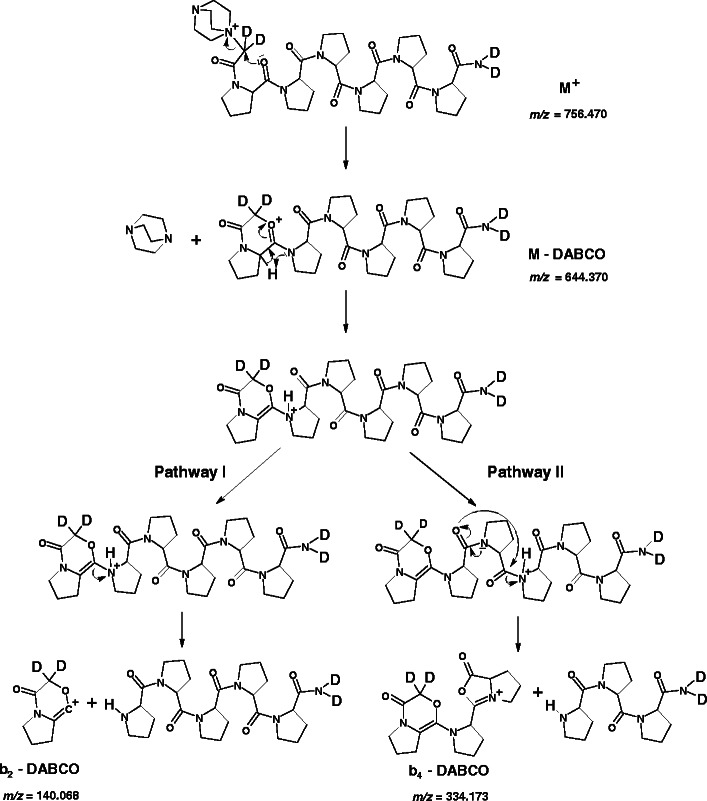
Possible pathways of [*b*_*2*_ – DABCO] (Pathway I) and [*b*_*4*_ - DABCO] (Pathway II) ions formation from **(d**_**4**_ **-NH,C** ^**α**^ **H)-4b** precursor ion, proposed on the basis of ESI-MS/MS spectrum presented in the Figure 2a. Theoretical *m/z* values are given

In the fragmentation pathway for [*b* – DABCO] ion formation, a key role plays a mobile proton generated due to a proline α-hydrogen shifting. The electron pair transfer of the bond between bicyclic structure and an amide nitrogen of the second proline residue in the sequence resulted in a fragment [*b*_*2*_ - DABCO] formation (Scheme 4, pathway I). A similar fragmentation pathway was proposed previously for *b*-type ions produced in the cleavage of the amide bond N-terminal to proline [15].

A mobile proton can also migrate along the peptide backbone and participate in charge directed fragmentation. According to the b_x_-y_z_ pathway, the fragmentation is initiated by a proton transfer to the nitrogen atom of the cleaved amide bond [3]. Then a nucleophilic attack by the oxygen of the N-terminal neighbor amide bond on the carbon center of the protonated amide bond leads to formation of an oxazolone structure of [*b* – DABCO] ion (Scheme 4, pathway II). Although during [*b* – DABCO] fragment formation hydrogen scrambling may occur, all mobile hydrogens and deuteriums remain in the neutral fragments. Thus, in the MS/MS spectra single peaks were observed for these fragment ions.

The fragmentation pathways proposed for oligoproline QAS-peptides involve α, β, γ or δ hydrogen shifting during fragment ion formation. For *N,N,N*-triethylammoniumacetyl derivatives the generation of mobile proton due to Hofmann elimination of ethylene molecule from QAS group is also possible [25]. The hydrogen transferred onto nitrogen atom is mobile and can undergo hydrogen scrambling with deuterons located on the C-terminal amide moiety. The mobilization of hydrogen initiates the cleavage of peptide bonds in the peptide ion. From this point of view the fragmentation mechanism shows significant similarity to that observed for the typical CID of protonated peptides which explains the formation of *y* and *b* ions.

## Conclusions

The fragmentation of QAS-peptide derivatives lacking easily mobilizable protons was examined with the aid of deuterium-labeled analogs. The obtained results indicate that the first step of fragmentation consists of elimination of tertiary amine molecule. This process generates positively charged center localized on the oxygen atom, which consequently leads to shift and mobilization of a hydrogen localized at α carbon. The hydrogen transferred onto nitrogen atom is mobile and can undergo hydrogen scrambling with deuteriums located in the C-terminal amide moiety. The mobilization of the protons enables facile amide bond cleavage and, thus, the formation of b and y ions. The y-type ions formation in oligoproline QAS-peptide requires generation of two mobile protons. The additional proton must be transferred from γ- or δ-carbon atoms of proline residue. Therefore, the fragmentation of oligoproline QAS-peptide derivatives involves the unusual mobilization of hydrogens localized at α- and γ- or δ-carbon atoms in the amino acid side chains. To the best of our knowledge, the fragmentation pathways of peptides lacking any mobile protons have not been reported. The study of this dissociation pattern highlights the unusual proline residue behavior during MS/MS experiments of peptides.

## Electronic supplementary material

Below is the link to the electronic supplementary material.

ESM 1 (DOC 706 kb)
