# Supplementary material for: Gas-Phase Fragmentation of Oligoproline Peptide Ions Lacking Easily Mobilizable Protons
Source: J Am Soc Mass Spectrom. 2013 Apr 23;24(6):846–56. doi: 10.1007/s13361-013-0585-1 (PMC3650240; doi:10.1007/s13361-013-0585-1)
Supplement: Supplementary file 1 — (DOC 706 kb) [file 13361_2013_585_MOESM1_ESM.doc]

Supplementary data

Journal of the American Society for Mass Spectrometry

**Gas-phase fragmentation of oligoproline peptide ions lacking easily mobilizable protons**

Magdalena Rudowska, Robert Wieczorek, Alicja Kluczyk, Piotr Stefanowicz, Zbigniew Szewczuk

*Faculty of Chemistry, University of Wrocław, Wrocław, Poland*

Running title: CID of peptides lacking mobile protons

Correspondence to: Z. Szewczuk, Faculty of Chemistry, University of Wrocław
F. Joliot-Curie 14, 50-383 Wrocław, Poland

E-mail: zbigniew.szewczuk@chem.uni.wroc.pl

Table 1S. Mass analysis of the synthesized QAS-peptide derivatives.

| **Nr** | **Sequence of QAS-peptide** | **M^+^_found_**  ***m/z^a^*** | **M^+^_calculated for formula_** |
| --- | --- | --- | --- |
| **1a** | Et_3_N^+^-CH_2_CO-(Pro)_3_-NH_2_ | 450.306 | 450.307; C_23_H_40_N_5_O_4_ |
| **1b** | DABCO^+^-CH_2_CO-(Pro)_3_-NH_2_ | 461.286 | 461.287; C_23_H_37_N_6_O_4_ |
| **2a** | Et_3_N^+^-CH_2_CO-(Pro)_4_-NH_2_ | 547.358 | 547.360; C_28_H_47_N_6_O_5_ |
| **2b** | DABCO^+^-CH_2_CO-(Pro)_4_-NH_2_ | 558.338 | 558.340; C_28_H_44_N_7_O_5_ |
| **3a** | Et_3_N^+^-CH_2_CO-(Pro)_5_-NH_2_ | 644.412 | 644.413; C_33_H_54_N_7_O_6_ |
| **3b** | DABCO^+^-CH_2_CO-(Pro)_5_-NH­_2_ | 655.393 | 655.393; C_33_H_51_N_8_O_6_ |
| **4a** | Et_3_N^+^-CH_2_CO-(Pro)_6_-NH_2_ | 741.466 | 741.466; C_38_H_61_N_8_O_7_ |
| **4b** | DABCO^+^-CH_2_CO-(Pro)_6_-NH_2_ | 752.446 | 752.445; C_38_H_58_N_9_O_7_ |
| **5a** | Et_3_N^+^-CH_2_CO-(Pro)_3_-OCH_3_ | 465.308 | 465.307; C_24_H_41_N_4_O_5_ |
| **5b** | DABCO^+^-CH_2_CO-(Pro)_3_-OCH_3_ | 476.287 | 476.287; C_24_H_38_N_5_O_5_ |
| **6a** | Et_3_N^+^-CH_2_CO-(Pro)_4_-OCH_3_ | 562.360 | 562.360; C_29_H_48_N_5_O_6_ |
| **6b** | DABCO^+^-CH_2_CO-(Pro)_4_-OCH_3_ | 573.339 | 573.339; C_29_H_45_N_6_O_6_ |
| **7a** | Et_3_N^+^-CH_2_CO-(Pro)_5_-OCH_3_ | 659.411 | 659.413; C_34_H_55_N_6_O_7_ |
| **7b** | DABCO^+^-CH_2_CO-(Pro)_5_-OCH_3_ | 670.389 | 670.392; C_34_H_52_N_7_O_7_ |
| **8a** | Et_3_N^+^-CH_2_CO-(Pro)_6_-OCH_3_ | 756.461 | 756.465; C_39_H_62_N_7_O_8_ |
| **8b** | DABCO^+^-CH_2_CO-(Pro)_6_-OCH_3_ | 767.440 | 767.445; C_39_H_62_N_7_O_8_ |

^a^ *m/z* values are presented for the monoisotopic ions.

Figure 1S. The strategies of solid phase synthesis of QAS-peptides. Aaa = amino acid residue, MW = microwave irradiation

Figure 2S. ESI-MS/MS spectra of the M^+^ molecular ions of peptide **1a** (A)**, 2a** (B), **3a** (C) and **4a** (D). The collision energy was set at 21 eV (A), 26 eV (B), 30 eV (C) and 35 eV (D).

Figure 3S. ESI-MS/MS spectra of the M^+^ molecular ions of peptide **1b** (A), **2b** (B), **3b** (C) and **4b** (D). The collision energy was set at 25 eV (A), 28 eV (B), 34 eV (C) and 40 eV (D).

Figure 4S. ESI-MS/MS spectra of the M^+^ molecular ions of peptide **5a** (A)**, 6a** (B), and **7a** (C). The collision energy was set at 23 eV (A), 27 eV (B) and 30 eV (C).

Figure 5S. ESI-MS/MS spectra of the M^+^ molecular ions of peptide **5b** (A), **6b** (B), and **7b** (C). The collision energy was set at 25 eV (A), 30 eV (B) and 35 eV (C).

Figure 6S. ESI-MS/MS spectra of the M^+^ molecular ion of peptide **8a**, recorded in various collision energy: 10 eV (A), 20 eV (B), 30 eV (C) and 35 eV (D).

Figure 7S. ESI-MS/MS spectra of the M^+^ molecular ion of peptide **8b**, recorded in various collision energy: 10 eV (A), 20 eV (B), 30 eV (C) and 35 eV (D).

Figure 8S. ESI-MS/MS spectra of deuterated peptides: **(d_4_-NH,C^α^H)-1a** (A), **(d_4_-NH,C^α^H)-2a** (B), **(d_4_-NH,C^α^H)-3a** (C) and **(d_4_-NH,C^α^H)-4a** (D). The peaks of representative *b* and *y* fragments are shown in insets. The collision energy was set at 22 eV (A), 25 eV (B), 30 eV (C) and 33 eV (D).

Figure 9S. ESI-MS/MS spectra of deuterated peptides: **(d_4_-NH,C^α^H)-1b** (A), **(d_4_-NH,C^α^H)-2b** (B) and **(d_4_-NH,C^α^H)-3b** (C). The peaks of representative *b* and *y* fragments are shown in insets. The collision energy was set at 25 eV (A), 29 eV (B) and 33 eV (C).

Figure 10S. ESI-MS/MS spectra of deuterated peptides: **(d_2_-C^α^H)-5a** (A), **(d_2_-C^α^H)-6a** (B), **(d_2_-C^α^H)-7a** (C) and **(d_2_-C^α^H)-8a** (D). The peaks of representative *b* and *y* fragments are shown in insets. The collision energy was set at 23 eV (A), 26 eV (B), 30 eV (C) and 34 eV (D).

Figure 11S. ESI-MS/MS spectra of deuterated peptides: **(d_2_-C^α^H)-5b** (A), **(d_2_-C^α^H)-6b** (B) and **(d_2_-C^α^H)-7b** (C). The peaks of representative *b* and *y* fragments are shown in insets. The collision energy was set at 25 eV (A), 28 eV (B) and 32 eV (C).

Figure 12S. ESI-MS/MS spectrum of peptide (**d_4_-NH,C^α^H)-4b**, recorded for collision energy 25 V. The peak corresponding to a fragment [M - DABCO - CD_2_CO] is shown in inset.


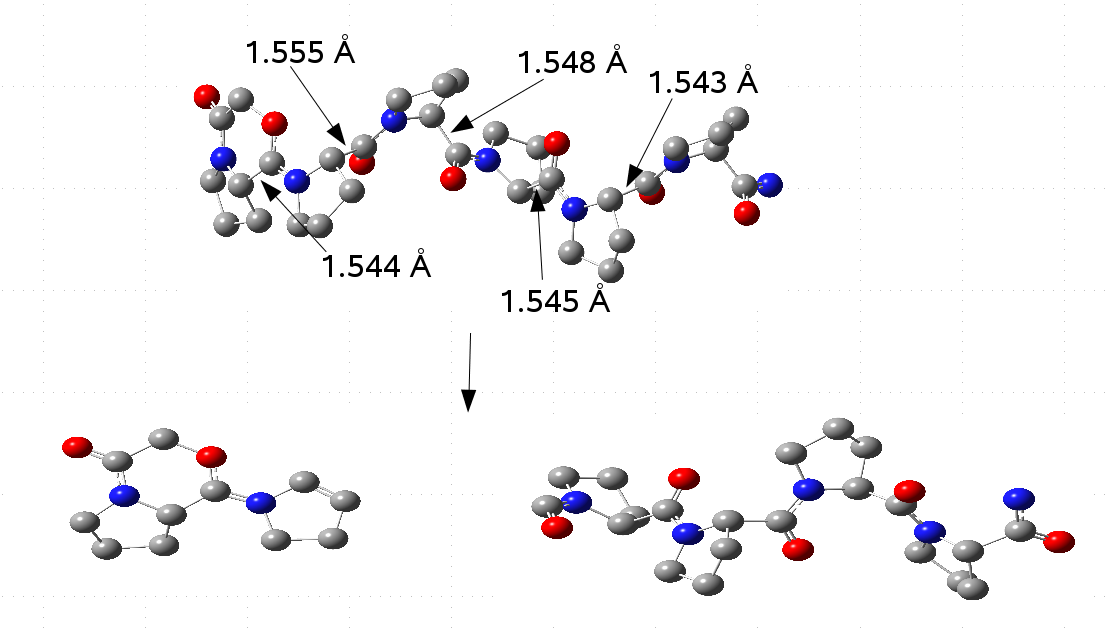


Figure 13S. Theoretical calculated C-C bonds set of **(d_4_-NH,C^α^H)-4b** backbone and preferred fragmentation products. Presented graphics contains heavy atoms exclusively. Presented structures of substrate and products are fully optimized at DFT level of theory.
